# Application of Multiscale Entropy in Assessing Plantar Skin Blood Flow Dynamics in Diabetics with Peripheral Neuropathy

**DOI:** 10.3390/e20020127

**Published:** 2018-02-15

**Authors:** Fuyuan Liao, Gladys L. Y. Cheing, Weiyan Ren, Sanjiv Jain, Yih-Kuen Jan

**Affiliations:** 1 Department of Biomedical Engineering, Xi’an Technological University, Xi’an 710021, China; 2 Department of Rehabilitation Sciences, The Hong Kong Polytechnic University, Hong Kong 999077, China; 3 Rehabilitation Engineering Lab, Department of Kinesiology and Community Health, University of Illinois at Urbana-Champaign, 1206 South Fourth Street, MC-588, Champaign, IL 61820, USA; 4 Beijing Advanced Innovation Center for Biomedical Engineering, School of Biological Science and Medical Engineering, Beihang University, Beijing 100083, China; 5 Department of Physical Medicine and Rehabilitation, Carle Hospital, Urbana, IL 61801, USA

**Keywords:** multiscale entropy, regularity, skin blood flow, diabetic foot ulcers

## Abstract

Diabetic foot ulcer (DFU) is a common complication of diabetes mellitus, while tissue ischemia caused by impaired vasodilatory response to plantar pressure is thought to be a major factor of the development of DFUs, which has been assessed using various measures of skin blood flow (SBF) in the time or frequency domain. These measures, however, are incapable of characterizing nonlinear dynamics of SBF, which is an indicator of pathologic alterations of microcirculation in the diabetic foot. This study recruited 18 type 2 diabetics with peripheral neuropathy and eight healthy controls. SBF at the first metatarsal head in response to locally applied pressure and heating was measured using laser Doppler flowmetry. A multiscale entropy algorithm was utilized to quantify the regularity degree of the SBF responses. The results showed that during reactive hyperemia and thermally induced biphasic response, the regularity degree of SBF in diabetics underwent only small changes compared to baseline and significantly differed from that in controls at multiple scales (*p* < 0.05). On the other hand, the transition of regularity degree of SBF in diabetics distinctively differed from that in controls (*p* < 0.05). These findings indicated that multiscale entropy could provide a more comprehensive assessment of impaired microvascular reactivity in the diabetic foot compared to other entropy measures based on only a single scale, which strengthens the use of plantar SBF dynamics to assess the risk for DFU.

## 1. Introduction

Diabetic foot ulcer (DFU) is a common complication of diabetes mellitus [1,2] and a major cause of hospitalization and non-traumatic lower-extremity amputations among people with diabetes [3]. The yearly and lifetime incidences of DFU are estimated to be about 2% and 15–25%, respectively [1], and the amputation rates in diabetics were reported to be 10–30 times higher than in the non-diabetic population [4,5]. Since treatment of DFUs is challenging and its economic burden is high [1,2], prevention of DFUs is highly important and has been recognized as a priority of diabetes healthcare [2].

The formation and development of DFUs involve a number of risk factors, among which peripheral neuropathy and peripheral arterial disease are crucial factors [1,2]. Diabetic peripheral neuropathy induces a series of pathologic alterations in the foot such as a loss of protective sensation for detecting mechanical stresses and/or trauma, foot deformities may result in elevated plantar pressure, and dryness of the skin that contributes to skin breakdown [6]. These alterations increase the risk of trauma and subsequent ulceration [6]. Also, it has been found that the majority of foot ulcers involve tissue ischemia [3], which is thought to be a major factor of the development of DFUs [6]. In the diabetic foot, impaired vasodilatory response to repetitive plantar pressure during walking is a main cause of plantar tissue ischemia [7], while impaired vasodilatory response to elevated temperature of the foot can aggravate tissue ischemia, because elevated skin temperature increases the metabolic demands of local cells and tissues, thus requiring an increase in skin blood flow (SBF) to meet the metabolic demands. Therefore, quantification of SBF responses to loading pressure and thermal stresses may be a reasonable way to assess the risk of DFU [7,8].

Traditionally, SBF response to mechanical stress is quantified using time-domain parameters such as normalized mean blood flow (divided by basal blood flow) during mechanical stress and measures of hyperemia [7,9]; SBF response to local heating is quantified using normalized first peak, nadir, and second peak (divided by basal blood flow) [7,9]. Moreover, wavelet-based spectral analysis has been utilized to investigate the underlying mechanisms of the responses [7]. It has been found that blood flow oscillations (BFO) in the human skin contain six characteristic frequency components in the frequency interval 0.005–2 Hz [10,11]. Two components with higher frequencies are originated from cardiac activity (0.4–2 Hz) and respiration (0.15–0.4 Hz), respectively. The other four components are associated with the myogenic activity of vascular smooth muscle (0.05–0.15 Hz), the neurogenic activity of the vessel wall (0.02–0.05 Hz), nitric oxide-related endothelia activity (0.0095–2 Hz), and nitric oxide-independent endothelia activity (0.005–0.0095 Hz), respectively. Jan et al. [7] investigated SBF responses at the first metatarsal head of diabetics induced by pressure loading and local heating, and showed an attenuated myogenic component during reactive hyperemia and attenuated metabolic, neurogenic, and myogenic components in response to local heating compared to healthy controls. 

Although time-domain parameters provide direct features of the SBF responses, and wavelet analysis provides a mean for characterizing the state of the regulatory mechanisms of SBF during the responses, they are unable to characterize the nonlinear features of BFO [12], which are the structural features of BFO, e.g., complexity and self-similarity, rather than the magnitude of variability. There is evidence that altered nonlinear properties of physiological signals are an indicator of pathologic changes in the physiologic system [8,13,14,15]. In our previous study [8], we utilized a modified sample entropy method [13] to quantify the regularity degree of SBF in diabetics, and showed promising results. However, because we used a fixed parameter, i.e., the time delay between neighboring data points of the sequences to be compared, we were unable to gain insight into how the regularity degree of BFO changed with time scales, which is likely associated with the homogeneity degree of the combination of characteristic frequency components. Therefore, the objective of the current study was to investigate the regularity degree of SBF responses at the first metatarsal head of diabetics induced by loading pressure and thermal stress at multiple scales and how regularity degree changed at various scales. We hypothesized that the transition of regularity degree of BFO could reflect microvascular dysfunction in diabetics.

## 2. Methods

### 2.1. Participants and Data Collection

Eighteen people with type 2 diabetes and peripheral neuropathy (13 men and 5 women) and eight healthy controls (four men and four women) were recruited into this study. The demographic data of participants and experimental protocols were presented in our previous publication [8]. Briefly, the diabetic subjects had a mean age (standard deviation, SD) of 48.5 (9.4) years, body mass index (BMI) of 28.3 (7.1) kg/m^2^, duration of diabetes of 15.2 (5.1) years, and HbA_1c_ level of 7.8 (0.9) %. Each of them suffered from peripheral neuropathy and had a history of foot ulcers. The healthy controls had a mean age (SD) of 21.8 (2.4) years and BMI of 25.8 (3.3) kg/m^2^. This study was approved by a university institutional review board (IRB #14707).

**Figure 1:**
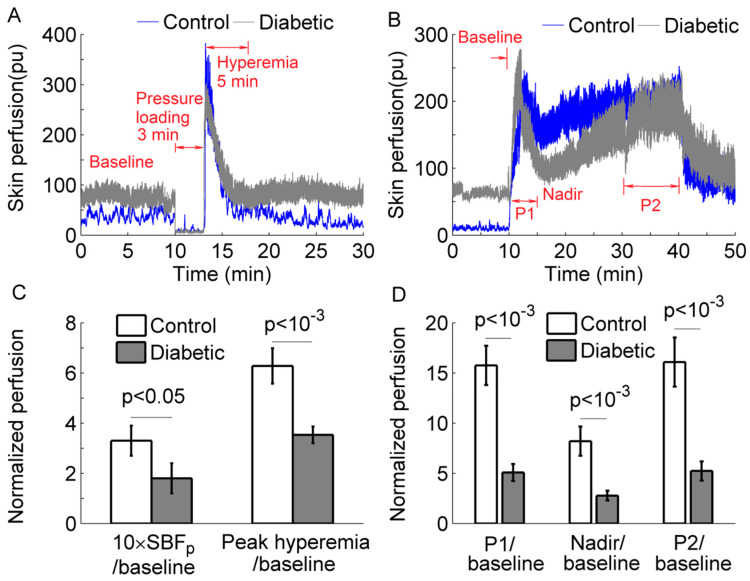
(**A**,**B**) Skin blood flow (SBF) responses to a loading pressure of 300 mmHg (**A**) and local heating (**B**) at the first metatarsal head of a diabetic subject and a healthy control. pu, perfusion unit. (**C**) Normalized SBF (divided by basal SBF) during the loading period and normalized peak hyperemia in two groups. (**D**) Normalized SBF during P1, nadir, and P2 in two groups. Data are represented as mean ± standard error. The differences in SBF between two groups were examined using Mann–Whitney U tests.

The experiments were conducted in a research laboratory with the room temperature being maintained at 24 ± 2 °C. Prior to any test, the subject was acclimated to the room temperature for at least 30 min. Then, the subject lay in a supine position and underwent two experiments. The first experiment was aimed to examine SBF response to locally applied pressure at the first metatarsal head. SBF and skin temperature were measured with a sampling rate of 32 Hz using a Laser Doppler flowmetry (PeriFlux 5001, Perimed, Ardmore, PA, USA), and a probe with heating function (Probe 415–242, Perimed). This protocol included a 10-min basal measurement, followed by a 3-min period during which a 300 mmHg pressure was applied to the probe via a computer-controlled indenter [7], and a 17-min recovery period. Figure 1A shows SBF responses in a diabetic subject and a healthy control; Figure 1C shows normalized SBF during the loading period and normalized peak hyperemia in two groups.

The second protocol was aimed to examine SBF at the first metatarsal head in response to local heating. This protocol included a 10-min basal measurement, a 30-min heating period during which the skin was heated to 42 °C in 2 min and the temperature was maintained at that level, followed by a recovery period lasting 10 min. Figure 1B shows SBF responses in a diabetic subject and a healthy control. This response was quantified using three indices: first peak (P1), nadir, and second peak (P2) divided by basal blood flow. Because SBF exhibited a plateau during the later period of heating (Figure 1B), the mean value of SBF during the last 10 min was defined as P2. Figure 1D shows the results of normalized P1, nadir, and P2 in two groups.

### 2.2. Sample Entropy and Its Derivatives 

**Figure 2:**
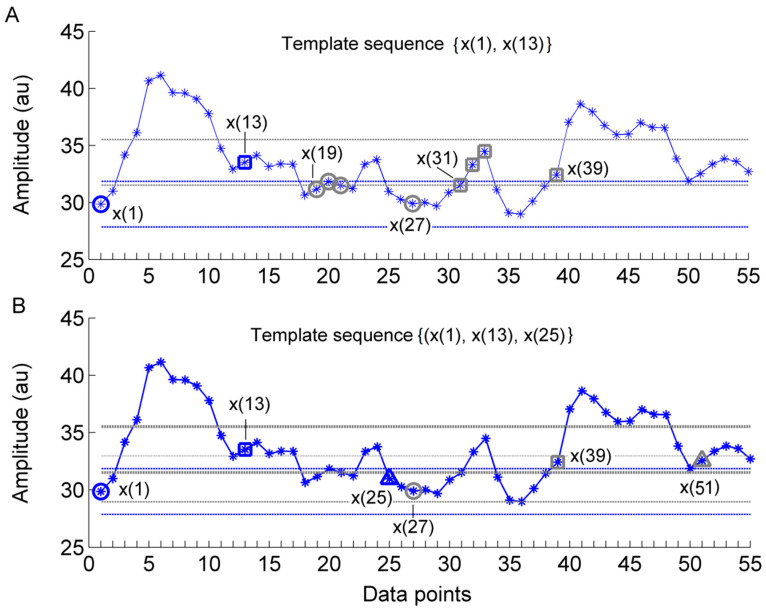
Illustration of a main procedure for calculating the modified sample entropy ($E_{MS}$) in the case of m = 2, τ = 12, and r=0.2×SD (the standard deviation of the series). (**A**) For a m-component template sequence $x_{m}^{\tau }(i)$ = {x(1), x(13)}, i.e., i = 1, there are four sequences $x_{m}^{\tau }(j)$ = {x(19,x(31)), {x(20),x(32)}, {x(21),x(33)}, and {x(27),x(39)}, i.e., j = 19, 20, 21, and 27, satisfying $d[x_{m}^{\tau }(i),x_{m}^{\tau }(j)]<r$, |j−i|>τ. This procedure is repeated for the next template vector $x_{m}^{\tau }(i)$ = {x(2),x(14)}, i.e., i=2, and so on. The dotted horizontal lines around data points x(1) and x(13) represent x(1)±r and x(13)±r, respectively. (**B**) The above procedure is repeated for all (m+1)-component sequences $x_{m+1}^{\tau }(i)$, e.g., {x(1), x(13),x(25)}.

Sample entropy ($E_{s}(m,r,N)$) is defined as the negative natural logarithm of the conditional probability that two sequences of m points within a tolerance r remain within the tolerance at the next point [16]. A smaller (larger) value of $E_{s}$ indicates a higher degree of regularity (irregularity). It has been demonstrated that $E_{s}$ depends on the relationship between the frequency of the dominant oscillations and the sampling rate [13]. Oversampling may lead to misleading results, i.e., the obtained $E_{s}$ value does not reflect the regularity degree of the dominant oscillations [13]. To address this problem, we recently developed a modified sample entropy algorithm [13]. Its procedures are presented briefly as follows. Figure 2 illustrates a main procedure of the algorithm.

For a time series {x(i),i=1,…,N}, consider the m-point sequences:

(1) $$x_{m}^{\tau }(i)=\{x(i+k\tau ),0\leq k\leq m-1\},\;1\leq i\leq N-m\tau ,$$

where τ is a lag. The condition 1≤i≤N−mτ ensures that $x_{m+1}^{\tau }(i)$ exits for i=N−mτ. The distance between two sequences $x_{m}^{\tau }(i)$ and $x_{m}^{\tau }(j)$ is defined as

(2) $$d[x_{m}^{\tau }(i),x_{m}^{\tau }(j)]=\max\{|x(i+k\tau )-x(j+k\tau )|,0\leq k\leq m-1\},\;|j-i|>\tau .$$

For a given sequence $x_{m}^{\tau }(i)$, let $n_{i}$ be the number of $x_{m}^{\tau }(j)$ satisfying |j−i|>τ and $n_{i}^{m}(r)$ the numbers of $x_{m}^{\tau }(j)$ satisfying $d[x_{m}^{\tau }(i),x_{m}^{\tau }(j)]<r$, |j−i|>τ, where r is a tolerance, usually being set to be proportional to the SD of the time series. The constraint condition |j−i|>τ is aimed to reduce influence of the correlation on entropy estimation [13]. Thus, $C_{i}^{m}(r)=n_{i}^{m}(r)/n_{i}$ represents the probability that any sequence $x_{m}^{\tau }(j)$ is within r of $x_{m}^{\tau }(i)$, and $C^{m}(r)=\sum_{i=1}^{N-m\tau }C_{i}^{m}/(N-m\tau )$ represents the probability that any two sequences $x_{m}^{\tau }(i)$ and $x_{m}^{\tau }(j)$ are within r. Likewise, $C^{m+1}(r)$ represents the probability that any two sequences $x_{m+1}^{\tau }(i)$ and $x_{m+1}^{\tau }(j)$ are within r. The modified sample entropy is defined as:

(3) $$E_{ms}(m,r,\tau )=\lim_{N\to \infty }-\ln\frac{C^{m+1}(r)}{C^{m}(r)},$$

which is estimated by:

(4) $$E_{ms}(m,r,\tau ,N)=-\ln\frac{C^{m+1}(r)}{C^{m}(r)}.$$

In our previous study [13], we tested $E_{ms}$ using simulated time series and SBF data. The results indicated that $E_{ms}$ yielded consistent values for various sampling rates, but $E_{s}$ cannot [13].

**Figure 3:**
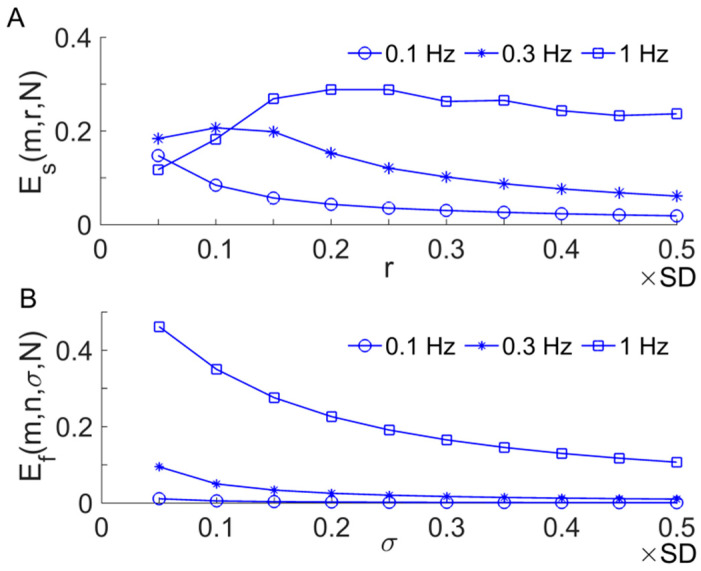
$E_{s}(m,r,N)$ (**A**) and $E_{f}(m,n,\sigma ,N)$ (**B**) of 0.1, 0.3, and 1 Hz sinusoidal signals sampled at a rate of 32 Hz. The parameters m = 2, n = 2, and N = 5760 were used. The parameter n = 2 was selected using an approach recommended by the authors who proposed $E_{f}$ [17].

Another representative derivative of $E_{s}$ is the fuzzy entropy $E_{f}(m,n,\sigma ,N)$ [17], which differs from $E_{s}$ in two aspects. First, when calculating the distance between two sequences $x_{m}(i)$ and $x_{m}(j)$, they are converted to have zero means. Second, the similarity between two sequences is quantified using an exponential function $\exp(-{\left(d_{ij}^{m}\right)}^{n}/\sigma )$, where $d_{ij}^{m}$ is the distance between them. It was reported that $E_{f}$ is more robust than $E_{s}$ when applying to short time series [17]. We tested the performance of $E_{f}$ and $E_{s}$ using sinusoidal signals with frequencies of 0.1, 0.3, and 1 Hz, respectively, and examined how they changed with increasing values of r (or σ). The frequencies of the sinusoidal signals are roughly equal to the central frequencies of myogenic, respiratory, and cardiac components of BFO, respectively; their length is equal to that of SBF signals during the pressure loading period (3 min, 5760 points). The parameters m= 2, n= 2 were used. The later was selected using an approach recommended by the authors who proposed $E_{f}$ [17]. The results showed that $E_{f}$ yielded very small values for 0.3 Hz and especially 0.1 Hz sinusoidal signals (Figure 3B). This suggests that when applied to SBF signals, $E_{f}$ may be unable to reflect altered dynamics of the low-frequency components of BFO, e.g., metabolic (~0.01 Hz), neurogenic (~0.03 Hz), and myogenic (~0.1 Hz) components.

### 2.3. Multi-Scale Entropy 

Despite $E_{s}$ being widely used to assess the complexity of time series, it is actually a measure of regularity, and this is the case for its various derivatives. Currently, complexity has not been well defined [18], and it is intuitively associated with “meaningful structural richness” [19]. A major problem of $E_{s}$ and its derivatives is that they yield the highest values for white noise, which is unpredictable but without structural complexity, and they may also yield higher values for physiological signals in health condition and lower values in pathological conditions [8,13]. In this context, several multiscale entropy (MSE) methods were introduced to quantify the regularity degree of a time series at multiple scales [20,21,22]. The first MSE method was proposed by Costa et al. [20], in which the original time series is divided into non-overlapping segments, and a new series is constructed using the average of each segment with its order being preserved. Then, $E_{s}$ is computed for each new series. This method, however, has been found to have several limitations. First, the procedure for constructing new series is similar to applying a low-pass filter to the original time series followed by a downsampling procedure. It has been found that the frequency response of the low-pass filter shows side lobes in the stop band, which lead to aliasing during the downsampling and thus produce artifacts [22]. Second, the SD of the new series likely decreases with increasing scales, whereas in the MSE algorithm, a constant tolerance (a constant proportion of the SD of the original time series) is used for all scales, resulting in decreasing entropy values with increasing scales. Finally, the length of the new series decreases rapidly with increasing scales, impeding reliable estimations of the entropy at large scales. The drawbacks of the MSE method and several improved algorithms have been discussed in Reference [23].

**Figure 4:**
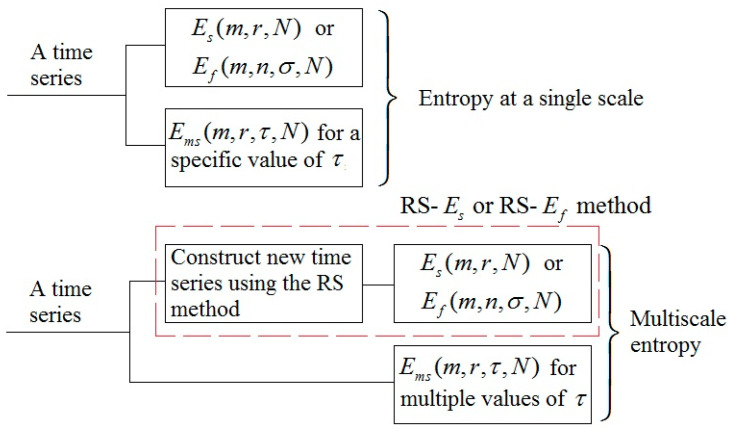
A flow diagram illustrating the relationships among the aforementioned entropy methods. RS, the reshape scale method [21].

Recently, a technique called reshape scale (RS) method was proposed to construct new time series from the original one [21]. Its main procedures are as follows. For a time series {x(i),i=1,…,N} and a scale factor τ, a new time series is constructed as:

(5) $$y^{\left(\tau \right)}=\{b_{1},b_{2},\ldots ,b_{\tau }\},$$

where $b_{i}=\{x(i),x(i+\tau ),\ldots ,x(i+k\tau )\}$, i=1,2,…,τ, and k is the maximal integer satisfying i+kτ≤N. Note that the length of $y^{\left(\tau \right)}$ is also N and when τ=1, it retrieves the original time series. Thus, a combination of the RS method and $E_{s}$ (or $E_{f}$) is a multiscale entropy method (denoted as RS-$E_{s}$ and RS-$E_{f}$, respectively). Figure 4 illustrates the relationships among the aforementioned entropy methods. Also, $E_{ms}(m,r,\tau ,N)$ is a multiscale entropy method when τ takes multiple values. the RS method is essentially similar to the first procedure of the $E_{ms}(m,r,\tau ,N)$ algorithm (Equation (1)). A main difference between them is that in the RS method the segment $b_{i}$, i=1,2,…,τ, can be randomly appended to other segments.

**Figure 5:**
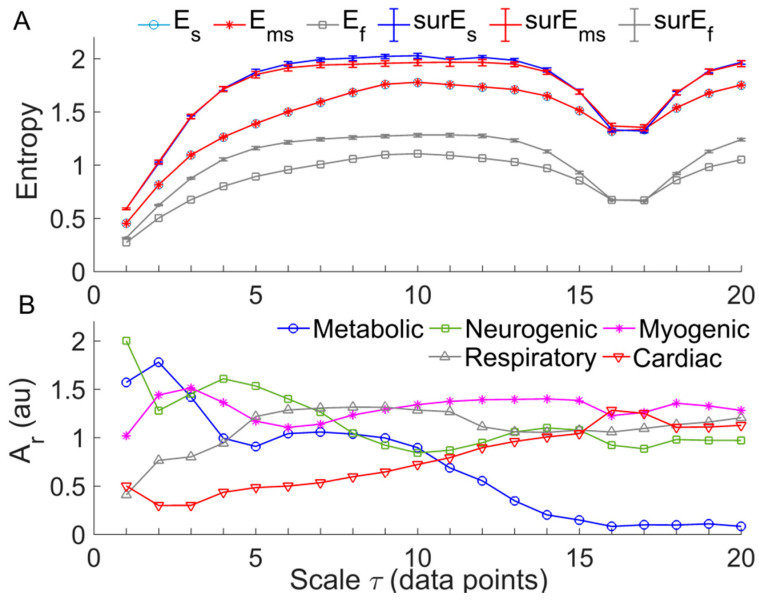
(**A**) Results of RS-$E_{s}$, RS-$E_{f}$, and $E_{ms}$ of a SBF signal from a diabetic subject (Figure 1A, 1–10 min). The following parameters were used: m = 2; r = 0.2 × SD (the generated signal at scale τ) for $E_{s}(m,r,N)$; r = 0.2 × SD (the original signal) for $E_{ms}(m,r,\tau ,N)$; n = 2; σ = 0.2 × SD (the generated signal at scale τ). The results of surrogate tests are presented as means ± standard errors. $surE_{s}$, $surE_{s}$, and $surE_{ms}$ refer to $E_{s}$, $E_{f}$, and $E_{ms}$ of phase-randomized surrogate data, respectively. For RS-$E_{s}$ and RS-$E_{f}$, 20 surrogate data sets were generated from the new signal at scale τ; for $E_{ms}$, 20 surrogate data sets were also generated from the original signal for each scale τ. (**B**) Relative wavelet amplitudes ($A_{r}$) of the metabolic, neurogenic, myogenic, respiratory, and cardiac frequencies of the generated signal at each scale τ using the RS method.

We tested the performance of three MSE methods, i.e., RS-$E_{s}$, RS-$E_{f}$), and $E_{ms}$, using SBF signals. The following parameters were selected: m=2; for RS-$E_{s}$, r was 0.2 × SD of the constructed signal; for $E_{ms}$, r was 0.2 × SD of the original signal; for RS-$E_{f}$, by using an approach recommended by the authors who proposed $E_{f}$ [17], we selected n= 2 and σ being 0.2 × SD of the constructed signal. The results showed that $E_{s}$ was almost identical to $E_{ms}$ at each scale, both of which monotonically increased at the scales from 1 to around 10 and then reach a plateau. The values of $E_{f}$ showed a similar trend but were much smaller than $E_{s}$ and $E_{ms}$. Figure 5A shows an example of the testing results. Further, we performed the following tests. For a given SBF signal, we computed $E_{s}$, $E_{f}$, and $E_{ms}$ for 20 phase-randomized surrogate data sets at each scale. When computing $E_{s}$ and $E_{f}$, surrogate data were generated from the new signal constructed by using the RS method, whereas when computing $E_{ms}$, surrogate data were generated from the original signal. The results showed that each of RS-$E_{s}$, RS-$E_{f}$, and $E_{ms}$ for surrogate data showed similar trend compared to that for the real data (Figure 5A). However, RS-$E_{f}$ yielded smaller differences between surrogate data and real data compared to RS-$E_{s}$ and $E_{ms}$. A possible reason is that $E_{f}$ is insensitive to structural changes of low-frequency components (see Figure 3B) caused by the phase randomization procedure. These testing results suggest that RS-$E_{f}$ has no superiority over RS-$E_{s}$ or $E_{ms}$ for assessing the complexity of BFO.

### 2.4. Multi-Scale Entropy of SBF Data

The above testing results (Figure 5A) indicate that $E_{ms}$ was almost identical to RS-$E_{s}$ and much larger than $E_{f}$ at each scale, while the transitions of $E_{ms}$ and RS-$E_{s}$ with increasing scales were similar to that of $E_{f}$. Therefore, we applied $E_{ms}$ to the SBF data collected from 18 diabetic patients and eight healthy controls. For the loading protocol, $E_{ms}$ was calculated for three segments of the SBF signal: baseline (1–10 min), loading period (11–13 min), and reactive hyperemia (a 5-min period following the peak hyperemia [8]); for the heating protocol, $E_{ms}$ was calculated for three segments of the SBF signal: baseline (1–10 min), P1 (a 5-min period following the beginning of the increase in SBF), and P2 (the last 10-min segment of the heating period). To eliminate the influences of possible ascending and/or descending trends as well as noise on $E_{ms}$, each data segment was filtered by decomposing it using the ensemble empirical mode decomposition method [24] and reconstructing a new signal from the intrinsic mode functions with frequencies between 0.0095 and 2 Hz [8]. Then $E_{ms}(m,r,\tau ,N)$ was computed for the filtered data at the scales from τ= 1 to 20. The parameters m= 2, r= 0.2 × SD were used.

### 2.5. Relative Wavelet Amplitude of BFO at Multiple Scales

To understand the underlying mechanisms responsible for the transition of $E_{ms}$, we applied wavelet analysis to the signals constructed from the original one using the RS method. For a constructed signal at scale τ, $y^{\left(\tau \right)}=\{y(i),i=1,\ldots ,N\}$ (Equation (5)), its continuous wavelet transform was defined as $w(s,t)=\int_{-\infty }^{+\infty }\psi_{s,t}(u)y(u)du$, where $\psi_{s,t}=\frac{1}{\sqrt{s}}\psi (\frac{u-t}{s})$, ψ(u) is the mother wavelet function, s is the scale corresponding to the central frequency of $\psi_{s,t}$, and t is time. In this study, we used the Morlet wavelet $\psi (u)=\pi^{-1/4}e^{-i\omega_{0}u}e^{-u^{2}/2}$ as the mother wavelet function, for which s is the reciprocal of the central frequency of $\psi_{s,t}$ when $\omega_{0}=2\pi$. Then we calculated the average amplitudes of the wavelet transform over time and over the frequency interval of five frequency components: metabolic (0.0095–2 Hz), neurogenic (0.02–0.05 Hz), myogenic (0.05–0.15 Hz), respiratory (0.15–0.4 Hz), and cardiac (0.4–2 Hz) components. Finally, the averaged wavelet amplitudes of the five frequency components were normalized (divided) by that of the frequency interval 0.0095–2 Hz to yield relative wavelet amplitudes ($A_{r}$). Figure 3B shows the $A_{r}$ values of the five frequency components of the SBF signal for calculating $E_{ms}$. A prominent feature of the changes in $A_{r}$ was that $A_{r}$ of the metabolic and cardiac components show persistent decrease and increase with increasing scales, respectively.

### 2.6. Statistical Analysis

The differences in multiscale entropy $E_{ms}$ and relative wavelet amplitude $A_{r}$ between two groups were examined using Mann–Whitney U test; the within-group differences in these measures were examined using Wilcoxon signed-rank test. These tests were performed using SPSS 16 (SPSS, Chicago, IL, USA).

## 3. Results

**Figure 6:**
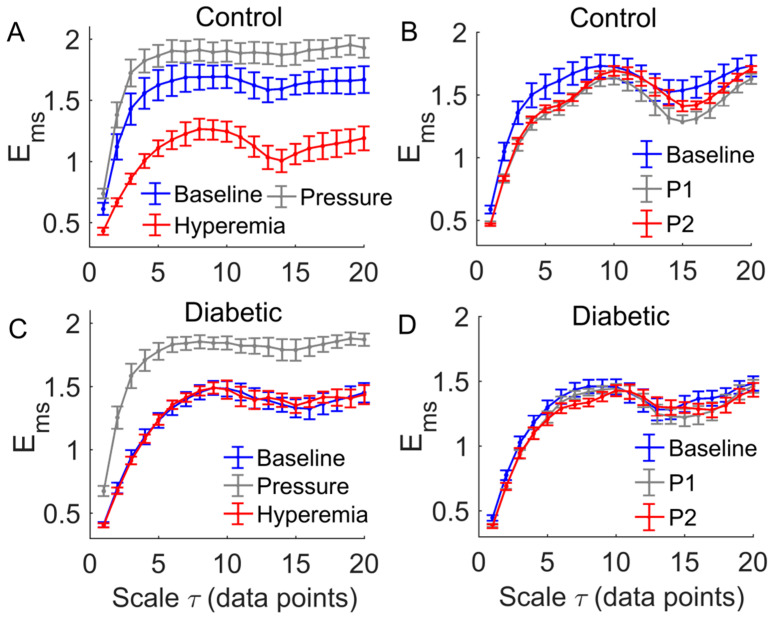
Within-group comparisons of $E_{ms}(m,r,\tau ,N)$. Data are represented as means ± standard errors. (**A**) In controls, comparison of $E_{ms}$ between hyperemia period and baseline yielded *p* < 0.05 at τ =1 to 7 and τ =16 to 18. (**B**) In controls, comparison of $E_{ms}$ between P1 and baseline yielded *p* < 0.05 at τ = 1 to 7 and τ =16 to 18, while comparison of $E_{ms}$ between P2 and baseline yielded *p* < 0.05 at τ =1 to 7. (**C**) In diabetics, comparison of $E_{ms}$ between the pressure period and baseline yielded *p* < 0.01 at all scales. (**D**) In diabetics, $E_{ms}(m,r,\tau ,N)$ yielded similar values during three periods of heating protocol.

**Figure 7:**
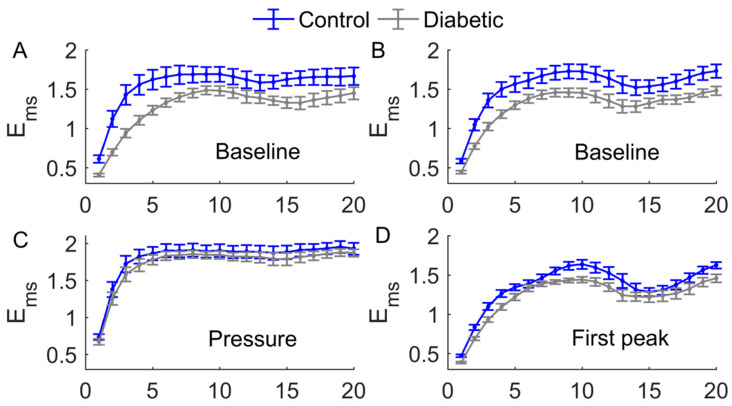

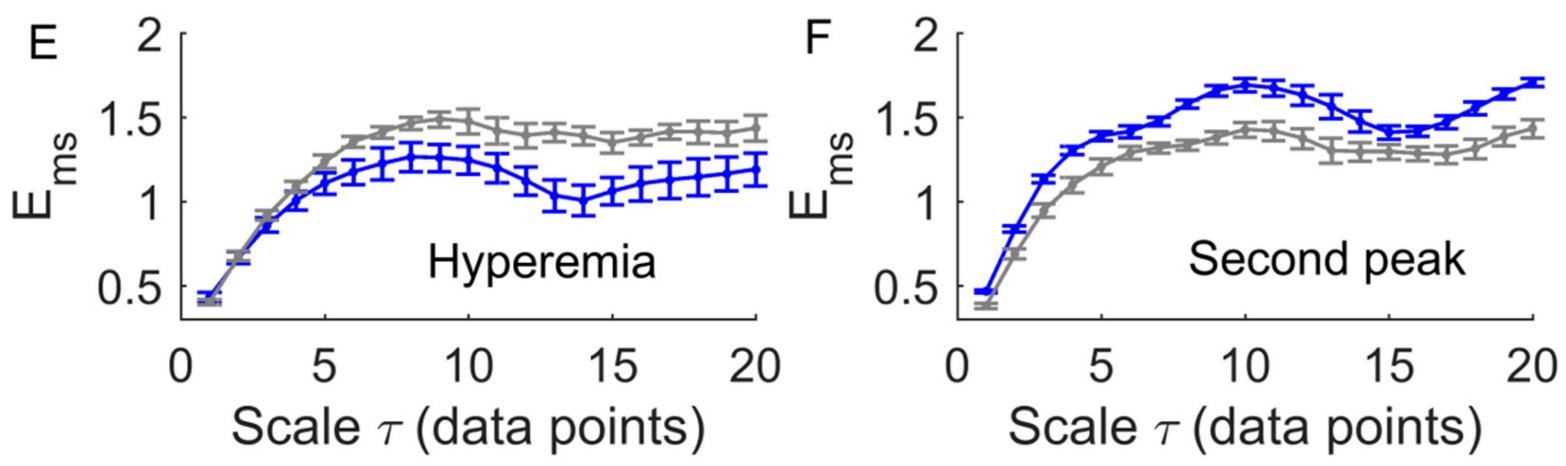
Comparisons of $E_{ms}(m,r,\tau ,N)$ between two groups. Data are represented as means ± standard errors. (**A**) During the baseline period of the pressure loading protocol, $E_{ms}$ in diabetics was significantly lower (*p* < 0.05) at the scales τ = 1 to 8 and τ = 14 to 16. (**B**) During the baseline period of the local heating protocol, $E_{ms}$ in diabetics was significantly lower (*p* < 0.05) at all scales. (**C**) During the loading pressure period, there was no significant difference between two groups. (**E**) During hyperemia, $E_{ms}$ in diabetics was significantly higher at τ = 13 to 18. (**D**) During P1, $E_{ms}$ in diabetics was significantly lower (*p* < 0.05) at τ = 1 to 4 and τ = 8 to 12. (**F**) During P2, $E_{ms}$ in diabetics was significantly lower (*p* < 0.05) at all scales.

A common feature of the multiscale entropy, $E_{ms}$, was that it rose with increasing scales at small scales and then reached a plateau (Figure 6 and Figure 7). Applied pressure resulted in a significant increase in $E_{ms}$ at all scales in diabetics (*p* < 0.01, Figure 6C) but not in controls (*p* > 0.05, Figure 6A). During reactive hyperemia, $E_{ms}$ in diabetics showed little change compared to baseline (Figure 6C), whereas in controls it showed a significantly decrease at the scales τ= 3 to 7 and τ= 10 to 20 (Figure 6A). Compared to controls, $E_{ms}$ in diabetics was significantly lower at the scales τ= 1 to 8 and τ= 14 to 16 during baseline (*p* < 0.05, Figure 7A,B), but was significantly higher at the scales τ= 13 to 18 during reactive hyperemia (*p* < 0.05, Figure 7E).

During thermally induced biphasic response, $E_{ms}$ in diabetics showed only small changes at all scales (Figure 6D), whereas in controls it significantly decreased at the scales τ= 1 to 7 and τ= 16 to 18 during P1 and at the scales τ= 1 to 7 during P2 (*p* < 0.05, Figure 6B). Compared to controls, $E_{ms}$ in diabetics was significantly lower at the scales τ= 1 to 4 and τ= 8 to 12 during P1 (*p* < 0.05, Figure 7D) and at all scales during P2 (*p* < 0.05, Figure 7F).

**Figure 8:**
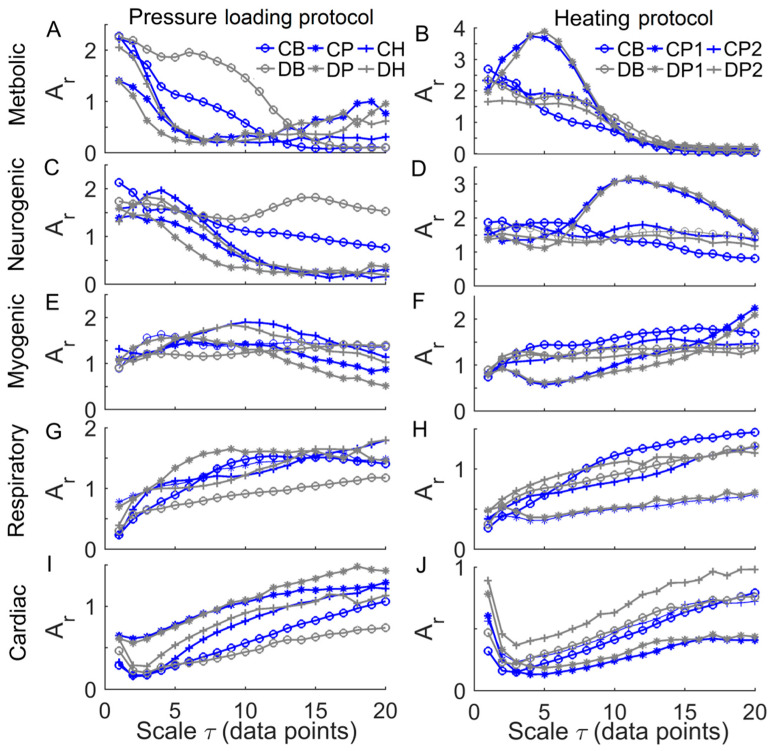
Relative wavelet amplitudes ($A_{r}$) of metabolic (**A**,**B**), neurogenic (**C**,**D**), myogenic (**E**,**F**), respiratory (**G**,**H**), and cardiac (**I**,**J**) components of blood flow oscillations (BFO) at multiple scales in response to loading pressure (left panels) and local heating (right panels). Data are represented as mean values in two groups. CB, CP, CH, CP1, and CP2 refer to baseline, loading pressure, hyperemia, first peak, and second peak periods in controls, respectively; DB, DP, DH, DP1, and DP2 refer to the same periods in diabetics.

Figure 8 shows the mean values of $A_{r}$ of the characteristic frequency components at multiple scales in two groups. A prominent feature was that $A_{r}$ of the metabolic component declined at small scales during baseline and the SBF responses except for thermally induced first peak (Figure 8A,B), while $A_{r}$ of cardiac component initially underwent a transient decrease followed by a sustained increase (Figure 8I,J). During thermally induced first peak, $A_{r}$ of metabolic component at small scales showed a pronounced increase followed by a substantial decrease (Figure 8B), while $A_{r}$ of neurogenic component exhibited similar changes but at larger scales (Figure 8D).

## 4. Discussion

The main findings of this study are as follows. First, during reactive hyperemia and the biphasic response induced by local heating, $E_{ms}$ in diabetics showed only small changes compared to baseline but in controls it underwent significant changes (Figure 6). As a consequence, $E_{ms}$ in diabetics significantly differed from that in controls at multiple scales (Figure 7). Second, during baseline and the SBF responses except for the pressure loading period, $E_{ms}$ at small scales exhibited different transitions between two groups (Figure 7). These findings indicated that multiscale entropy could provide a more comprehensive assessment of SBF dynamics compared to entropy measures on a single scale. Since the SBF data were recorded from the first metatarsal head, one of the most common sites of DFU, our findings support the use of nonlinear measures of SBF responses induced by mechanical and thermal stresses to assess the risk of DFU. 

In this study, we utilized a modified sample entropy algorithm [13] for computing multiscale entropy of BFO by varying the lag between neighboring data points of the sequences to be compared. We have demonstrated that this method yielded almost identical entropy values compared to the RS-$E_{s}$ method (Figure 5A), which is a combination of sample entropy [16], and a method for generating new signals from the original one, called reshape scale (RS) method [21]. We have also demonstrated that RS-$E_{f}$ has no superiority over $E_{ms}$ for assessing the complexity of BFO. Since computing $E_{f}$ is very time consuming, this measure may be more suitable for short series data, e.g., RR interval series.

A common feature of $E_{ms}$ of BFO was that it showed a rise with increasing scales at small scales, possibly including a rapid rise followed by a slow rise, and then reached a plateau (Figure 6 and Figure 7). To get an insight of the underlying mechanisms for this phenomenon, we calculated the relative wavelet amplitudes ($A_{r}$) of the characteristic frequency components of BFO and examined how they changed with increasing scales. A prominent feature of changes in $A_{r}$ was that $A_{r}$ of metabolic component deceased with increasing scales during baseline and the SBF responses except for thermally induced first peak (Figure 8A,B), while $A_{r}$ of the cardiac component initially showed a transient decrease and then underwent a sustained increase (Figure 8I,J). Therefore, we speculate that $E_{ms}$ of BFO reflects the homogeneity degree of the combination of the characteristic frequency components. Thus an augmentation of any frequency component, e.g., metabolic or cardiac components, will contribute to lowering $E_{ms}$. In this sense, any entropy measures on a single scale, e.g., sample entropy, only reflect the homogeneity degree of a specific combination of the characteristic frequency components. For instance, sample entropy (τ= 1) reflects the regularity degree of BFO where metabolic component and possibly cardiac component play a dominant role (Figure 8). In our previous study [8], because we used a fixed parameter τ= 12, we observed a significant influence of cardiac component on $E_{ms}$ during the thermally induced second peak.

Our results showed that during baseline and the SBF responses except for the pressure loading period, $E_{ms}$ in diabetics significantly differed from that in controls at multiple scales (Figure 7). Also, the transition of $E_{ms}$ in diabetics was different from that in controls. For instance, during baseline, $E_{ms}$ in diabetics showed a fairly rapid increase at the scales from τ= 1 to around 8 (Figure 7A,B), while in controls $E_{ms}$ showed a rapid increase at the scales from τ= 1 to 3, followed by a slow rise at the scales from τ= 3 to around 8 (Figure 7A,B). This distinct difference was partially due to different changes in $A_{r}$ of the metabolic component between two groups. As shown in Figure 8A,B, in diabetics $A_{r}$ of the metabolic component decayed slowly at small scales, whereas in controls it decayed rapidly. 

The association between $E_{ms}$ of BFO and $A_{r}$ of the characteristic frequency components can also be observed during the SBF responses. However, it should be kept in mind that $E_{ms}$ is a global measure of the structural properties of BFO, and may be influenced by all frequency components and the interactions among them [8]. $E_{ms}$ could reveal some global features of BFO that cannot be distinctively reflect by $A_{r}$ and vice versa. For example, we observed significantly higher values of $E_{ms}$ in diabetics at the scales from τ= 6 to 9 and from τ= 12 to 18 during reactive hyperemia (Figure 7C), but $A_{r}$ of each frequency component in diabetics was similar to that in controls (Figure 8, left panels). In contrast, during the thermally induced first peak, $E_{ms}$ in diabetics showed only small changes compared to baseline (Figure 6D), but the $A_{r}$ of each frequency component showed pronounced changes (Figure 8, right panels). Therefore, $E_{ms}$ and wavelet analysis could be mutually complementary in assessing SBF responses.

**Figure 9:**
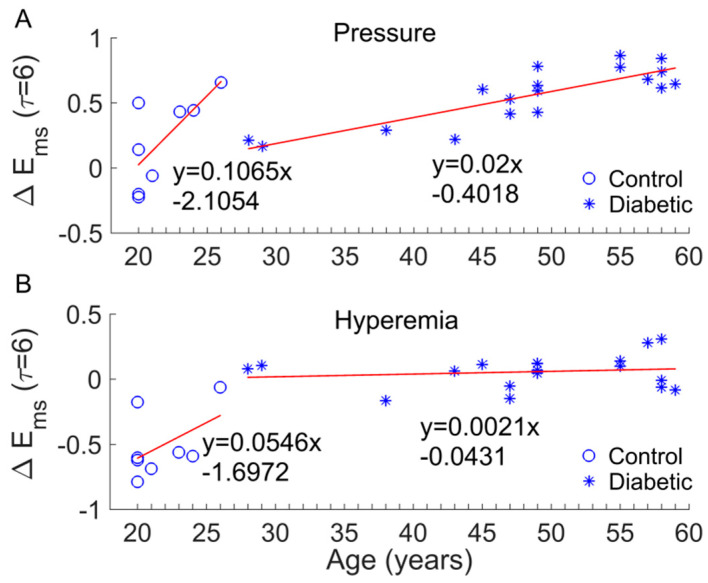
(**A**) Examination of the effect of age on changes in $E_{ms}$ during the pressure loading period compared to baseline (denoted as $\Delta E_{ms}$, scale factor τ = 6). $\Delta E_{ms}$ was significantly larger in diabetics than in controls (*p* < 0.05). (**B**) Examination of the effect of age on $\Delta E_{ms}$ (τ = 6) during hyperemia. The absolute value of $\Delta E_{ms}$ was significantly larger in controls (*p* < 0.001).

There are several limitations of this study. The sample size was small, which might impede the power of the statistical analysis. We did not have an age, sex, or BMI matched control. Factors such as age, sex, and BMI may affect our results. Since ageing can lead to altered dynamics of skin BFO [12,13], we focused on examining whether age significantly contributed to the results observed in this study. For example, we observed a significant increase in $E_{ms}$ during the pressure loading period compared to baseline in diabetics (Figure 6C) but not in controls (Figure 6A) and a significant decrease in $E_{ms}$ during hyperemia compared to baseline in controls (Figure 6A), but not in diabetics (Figure 6C). We thus examined whether the changes in $E_{ms}$ (denoted as $\Delta E_{ms}$) were related to age. Figure 9 shows the results in the case of τ= 6. On one hand, $\Delta E_{ms}$ was significantly different between two groups (*p* < 0.05 for pressure loading period and *p* < 0.001 for hyperemia). On the other hand, although $\Delta E_{ms}$ exhibited an increasing trend with age in both groups, the increasing rate (the slop of the fitting line) in diabetic group is much smaller than that in control group (Figure 9A,B). In particular, $\Delta E_{ms}$ in diabetic group during hyperemia was almost independent of age (Figure 9B). These results suggested that the significant difference in $\Delta E_{ms}$ between two groups was mainly attributed to impaired microvascular reactivity in the diabetic foot and that age has a marginal effect on BFO dynamics compared to diabetes. Future studies may follow up the development of DFU in a larger sample size with matched controls to validate our findings.

## 5. Conclusions

The present study indicated that during reactive hyperemia and the biphasic response induced by local heating, the regularity degree of SBF at the first metatarsal head of diabetics underwent only small changes compared to baseline, and significantly differed from that in healthy controls at multiple scales. On the other hand, the regularity degree of SBF in the diabetic foot displays distinctively different transitions compared to controls. This study suggests that multiscale entropy could provide a more comprehensive assessment of impaired microvascular reactivity in the diabetic foot compared to any entropy measures on a single scale and may be used to assess the risk for DFU.
